# The Effect of Juingong Meditation on the Theta to Alpha Ratio in the Temporoparietal and Anterior Frontal EEG Recordings

**DOI:** 10.3390/ijerph19031721

**Published:** 2022-02-02

**Authors:** Joohyun Kim, Miji Kim, Miran Jang, Junyeop Lee

**Affiliations:** 1 Department of Nursing, Collage of Nursing, Kangwon National University, Chuncheon 24341, Korea; joohkim@kangwon.ac.kr; 2 Hanmaeum Oriental Medicine Clinic, Anyang 13909, Korea; jimyoungin@naver.com; 3 Severance Biomedical Science Institute, College of Medicine, Yonsei University, Seoul 03722, Korea; mrjang421@gmail.com; 4 Yaksanae Oriental Medicine Hospital, Ansan 15549, Korea

**Keywords:** Juingong meditation, electroencephalography, brainwave, Buddhism

## Abstract

(1) Background: The effect of Juingong meditation on brainwave patterns has not been explored yet. This study aimed to study the changes in brainwave patterns produced by Juingong meditation, through electroencephalography (EEG) measurements. (2) Methods: The study included 23 participants from the Hanmaum Seon Center in Korea. EEG measurements were performed using InteraXon’s four-channel EEG measurement equipment, Muse. It measures EEG patterns in the temporoparietal and anterior frontal lobes. Brainwaves were measured in two different states: when Juingong meditation was practiced and when instructed mind wandering (IMW) was practiced. The EEG recordings were analyzed using the theta/alpha index. (3) Results: In the Juingong meditation state, the power of alpha was relatively higher than that of theta and these results were valid in the temporal parietal lobe channel. This indicates that relatively more alpha waves were induced in the temporal parietal lobe when Juingong meditation was practiced. (4) Conclusions: When Juingong meditation is practiced, the theta/alpha ratio changes without delay, which means that the practical effect of Juingong meditation on brainwave patterns is immediately apparent.

## 1. Introduction

Seon meditation, one of the practices of Buddhism, is a technique to calm the mind and contemplate the inner mind to reach the ultimate level of Buddhist practice. Seon meditation is known in the West as “meditation,” and its significance is gradually being recognized with the development of neuroscience [1]. Cardoso et al. define meditation as a procedure that includes (1) the use of a specific technique, (2) muscle relaxation (3) logic relaxation (4) it must necessarily be a self-induced state, and (5) the use of “self-focus” skills [2]. Meditation is one of the methods of practicing Buddhism and it has also been popularized in the non-religious sphere under the concept of mindfulness [3,4]. The origin of mindfulness is “Vipassana,” and the primary feature of this meditation is “choiceless awareness” [5] or “open monitoring” [6], which is a non-selective perception that focuses on recognizing actions such as one’s mind or breathing [7]. 

Seon Master Daehaeng is the founder of the Hanmaum Seon Center in Korea. The Hanmaum Seon Center is a Buddhist temple in Korea. Juingong meditation (Gwan) was defined by Seon Master Daehaeng and is a meditation practiced only at the Hanmaum Seon Center. Juingong is the fundamental mind with which each one of us is inherently endowed and which is directly connected to every single thing. “Juin” means the true doer, and “gong” means empty. Thus, Juingong is our true nature, our true essence, which is always changing and manifesting, and which has no fixed form or shape [8]. The Korean word “Gwan” literally means “watching,” but in terms of spiritual practice it means having faith and letting go to indulge in the observation of non-duality [8]. 

German psychiatrist and physiologist Hans Berger was the first to record electrical signals from the human brain and his first paper on EEG was titled “Über das Elektrenkephalogramm des Menschen” [9]. Among the methods for exploring changes in the brain during meditation, the most widely used is the electroencephalogram (EEG). In 1955, Das and Gastaut published the first study on the changes in the EEG that occur during practice. They reported changes in the alpha frequency of seven experts during meditation [10]. It has been found that the practice of contemplating one’s inner self leads to changes in brainwaves [11]. Recent studies have shown that theta waves [12,13] and high-frequency gamma waves above 100 Hz [14] increase during meditation. When the eyes are closed, the alpha waves dominate at the beginning, and when entering a more concentrated state, theta and delta waves become dominant [15]. Theta waves predominate in shallow sleep, hypnotic states, or at the boundary between consciousness and unconsciousness [16]. In brainwave studies on meditation, many results indicate that theta waves commonly increase in meditation states [12].

The theta to alpha ratio is an index calculated by using the theta power of the EEG as the numerator and alpha power as the denominator, and is derived from alpha–theta neurofeedback training [17]. Changing an EEG pattern using a feedback technique is known as neurofeedback. In the Alpha–Theta protocol, which focuses on relaxation therapy, the theta to alpha ratio is higher when the alpha–theta feedback training is performed. Alpha–theta feedback training is derived from the alpha–theta feedback protocol. This training is effective for post-traumatic stress disorder [18] or alcoholism [19], etc. The purpose of alpha–theta feedback is to train participants to increase theta versus alpha waves while they are not asleep. This leads to a state of deep relaxation similar to a meditative or quasi-hypnagogic state. Thus, alpha–theta feedback training aims to teach participants to enter a state that would normally be an unconscious state. This is because participants are trained to reach a stage of loss of consciousness or the beginning of sleep, reducing alpha waves and increasing theta waves [17]. The Alpha–Theta protocol continuously attempts to induce a state in which the theta wave is more dominant than the alpha wave in such a non-sleep state, or it increases the alpha and theta waves and reduces the beta wave [17]. Therefore, in the Alpha–Theta protocol, theta waves are dominant, and through deeper mental and physical relaxation, the stimulation of the central nervous system is reduced to increase the effectiveness of treatment [17].

EEG findings on meditation are not the same for all studies. In some cases, studies show contradictory results [6,14]. However, a number of studies have shown that the frontal, temporal, and occipital regions of participants are different from those of the control group during meditation, and the power of theta is stronger than that of the control group in the band [20,21,22]. In particular, in mindfulness meditation, there was a difference between the experimental group and the control group for theta and alpha waves in the occipital and right temporal areas, and the theta waves for the experimental group showed significantly stronger power compared to the control group during meditation [11,23]. Most of the meditation-related EEG studies that showed an increase in theta waves were based on Vipassana mindfulness [12,13,24]. Even when EEG measurements were performed using different meditation methods, the increase in theta waves was highest in the Vipassana method [6].

There has been recent EEG studies on various practices including meditation to improve participants’ sense of spirituality. The results, of the research showed that religious environments [25] and age [26] affect the EEG in practice. In particular, regarding age, the results showed that older participants obtained a sense of spirituality sooner [26].

To date, there have been many studies on mindfulness in brainwave research on meditation. However, even though many practitioners of Juingong meditation have had various religious and healing experiences [27], these personal experiences are not scientifically proven, and thus, the effects are not well recognized. The effect of Juingong meditation on brainwave patterns has not been explored yet. Therefore, in this study, we aimed to conduct objective research on Juingong meditation by verifying what kind of brainwave changes occur in participants practicing it. In particular, we attempted to understand how the theta to alpha ratio value used in the Alpha–Theta protocol appears during Juingong meditation.

## 2. Materials and Methods

### 2.1. Study Design

This study used a quasi-experimental method. Participants were tested under both conditions (Juingong meditation and control condition). There are many ways to practice Juingong meditation. The method used in this study was to make the practitioner think in their mind, “Juingong! Prove that you exist!” while sitting down [28]. In Juingong meditation, practitioners can sit if they want to sit, stand if they want to stand, work if they want to work—all of these are accepted ways of practicing meditation. However, in our experiment, we had to measure EEG, so we used a sitting meditation method. “Sitting” meditation means that practitioners maintain a calm and steady mind while entrusting every single thing to Juingong, with the belief that Juingong is the source and destination of all things [28].

Therefore, the EEG was measured with the participants in a sitting position. We needed a contrasting state of mind for the control condition compared to Juingong meditation. The control condition adopted the same body posture as the Juingong meditation. We referenced Cahn et al. for the control conditions [6]. We asked participants in the control condition to recall autobiographical memories that did not involve emotions [6]. This is called instructed mind wandering (IMW). Through this method, we were able to create a control state that prevented participants in the control condition from unintentionally entering a meditational state [6].

### 2.2. Participants

Participants for this study were recruited from the Hanmaum Seon Center in Anyang City, Korea. None of the study participants received any financial compensation. The experiment was approved by the Kangwon National University Institutional Review Board (KWNUIRB-2019-01-003-002). Participants were recruited from 20 May to 18 June 2019, considering their age, years of meditation practice, and the presence of disease (e.g., mental illness such as schizophrenia, mental retardation). Prior to the experiment, all participants met and received detailed information on the content of the experiment, the experimental procedure, and the purpose of the experiment. In addition, they were told that EEG measurements did not harm the human body, and they could withdraw from the experiment at any time if they did not feel fit enough to participate or for any other reason. Participants filled out a questionnaire at the meeting prior to the experiment. Through this questionnaire, information on the health status of the participants, drugs consumed, diseases, age, sex, and the number of times they practiced Juingong meditation were collected. The inclusion/exclusion criteria were as follows.

Inclusion criteria

- provide valid informed consent prior to any study procedure
- male or female subjects age 18 to 80 years

Exclusion criteria

- acute or chronic disorders (cardio-vascular, respiratory, neurologic, psychiatric)
- drinking before experiment
- took psychotropic drugs or were judged to be physically and mentally unsuitable for EEG

After completing the questionnaire, all participants, research directors, and researchers signed the consent form for the experiment issued by the Kangwon National University Institutional Review Board. All information about the study participants was stored using codes (M1, M2, etc.) instead of names so that personal information remained anonymous, and all data were handled and recorded as codes. The EEG measurements took place from 1 July to 19 July 2020. Of the 23 participants in the study, 5 were male (21.7%) and 18 were female (78.3%). The minimum and maximum age of the participants was 26 years and 77 years, respectively, with an average age of 55.3 years (SD = 14.01). The duration of attendance at Hanmaum Seon Center was at least 3 years, with a maximum of 35 years, and an average of 19 years (SD = 8.38). “Duration of attendance” means the period from the first time that participants went to the Hanmaum Seon Center to learn Juingong meditation up to the present. The participants do not live in the Hanmaum Seon Center, they just go there to practice. The Hanmaum Seon Center is an educational institution that teaches Juingong meditation. Here, participants can learn Juingong meditation 1–2 times a week. They can also practice it at any time and in any place. Thus, the number of times that participants practice Juingong meditation may vary from person to person and cannot be counted.

### 2.3. Setting and Samples

The number of samples required for this study was calculated using the G*Power 3.1 program. It was analyzed by a paired comparison t-test before and after one state. In the G*power test, when “matched pairs, one-tailed, effect size 0.6, alpha error probability 0.05, power 0.80” was set, the total number of samples was 19; accordingly, 23 people were finally recruited, considering a dropout rate of 20%.

### 2.4. Procedure

In this study, the differences in the EEG patterns between the states of Juingong meditation and IMW were compared among the participants. For this, participant were required to practice three states of meditation. The first was a Juingong meditation state (A), which was to be seated and practice Juingong meditation. In the second state (B), Juingong meditation was not practiced, instead, the participants performed IMW. Here, participants adopted the same seated posture as in the first state, but did not practice meditation. Performing IMW prevented participants from entering the meditation state. In the third state, the participant relaxed their sitting position (C) and rested freely. In this case, participants took a break outside the EEG measurement space, and performed free movements such as stretching. 

**Figure 1:**
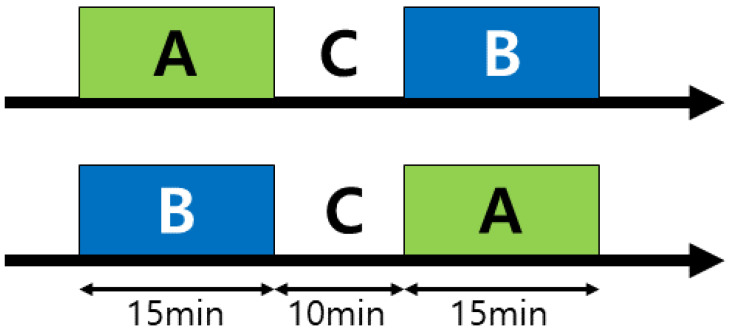
Process for measuring the practice of the participants. Both Juingong meditation (A) and IMW (B) lasted for 15 min. C was a 10 min break. Both states A and B were conducted in the same sitting posture. EEG was not measured during pre and post meditation or IMW. The EEG was measured only during the meditation (A) and IMW (B) states.

All study participants were randomly divided into two groups using CASIO’s Scientific calculator, FX-570ES PLUS-2′s random number generation function. This random division was intended to offset the effects of order. Participants performed all three states, A, B, and C. C was used as a break between A and B. A is Juingong meditation, and B is the IMW state. Participants did not know whether they would do A first or C first. The order was determined randomly. For example, if a person did A first, that person rested in the C state and then did B, and if he/she did B first, he/she rested in the C state and then did A (Figure 1). Participants sat on a cushion placed on the floor with their eyes closed during states A and B. During the EEG data collection, all lights in the room were turned off. EEG was not measured during the pre and post meditation and IMW stages. EEG was only measured in the meditation (A) and IMW (B) states.

### 2.5. Data Collection

EEG measurements were conducted in the meditation laboratory of the Hanmaum Science Institute. In order to maintain the objectivity of the study, the EEG measurement environment (place, time, and date) was maintained under the same or similar conditions, as far as possible. In this study, a four-channel EEG measurement device was used. The EEG measurement device was Muse from InteraXon, Canada, model MU-02, and serial number 2041-8TA2-8696. The device is a portable EEG measurement system powered by batteries and it has been widely used in other studies [29,30]. 

**Figure 2:**
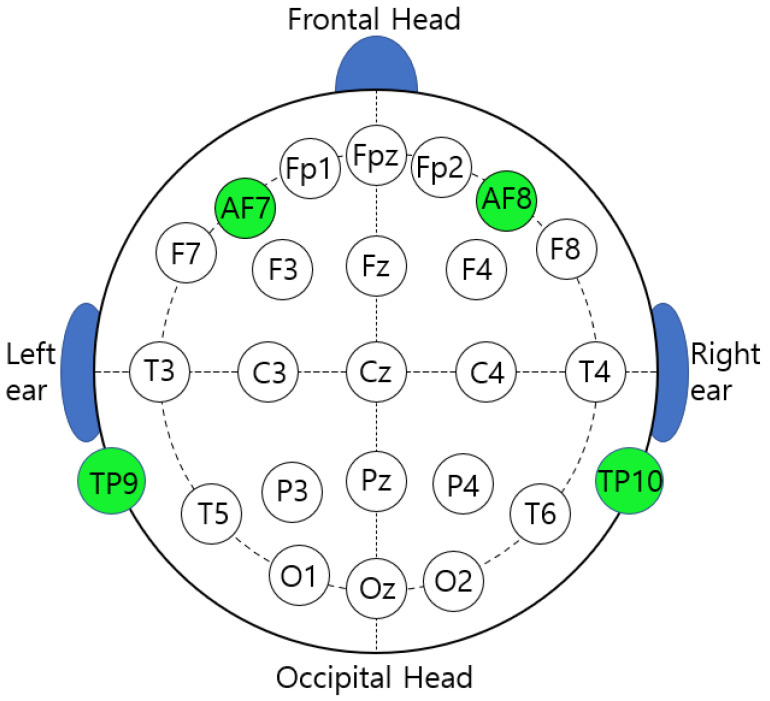
This figure shows the standard locations for measuring EEG as per 10–20 International standards. The green dots show the areas where the EEG was measured by the equipment used in this experiment. The FPz is located in the center and plays a role in determining the reference point of the EEG signal.

According to the 10–20 electrode method, four active electrodes were located at TP9 (temporoparietal), AF7 (anterior frontal), AF8 (anterior frontal), and TP10 (temporoparietal) (Figure 2). The Muse Research Resource Identifier (RRID) is SCR_014418. The sampling rate was 256 Hz and the reference channel was FPz. The EEG measurement site was an enclosed space where noise was blocked, and no electronic equipment other than the EEG measurement equipment was used. Before each experimental EEG measurement proceeded, the instrument was checked to ensure that the electromagnetic field measurement value was 0, using electromagnetic field measurement equipment (product name: IB005, company name: Lightcom, country of manufacture: China, serial number: H18069843). Since the forehead and ears of the participants were touched by the EEG sensor, before each EEG measurement, the equipment was carefully wiped with alcohol. The EEG measurements were performed by a researcher from the Yaksanae Oriental Hospital. 

### 2.6. Data Processing

Data analysis was performed in MATLAB (v9.5.0 R2018b, The MathWorks Inc., Natick, MA, USA) of the open-source software EEGLAB (v14.1.2, Swartz Center for Computational Neuroscience, University of California, San Diego, CA, USA). 

During the preprocessing, the EEGLAB software’s FIR Bandpass Filter (pop_eegfiltnew) was used to remove the EEG data (lower cut-off: 1 Hz, higher cut-off: 70 Hz). Second, 60 Hz line noise was removed using the pop_cleanline filter from the EEGLAB software. The EEG data collection channels were TP9, TP10, AF7, and AF8, and the sample rate was 256 Hz. In the frequency analysis (fast Fourier transform, FFT), short-time FFT (buffer size 1000 ms, no overlap) was applied. The EEG data was measured for 15 min for both the Juingong meditation state and IMW state.

The first 5 min was considered as the time required for the participants to attain a level of concentration, therefore, the data for the first 5 min in both the Juingong meditation state and IMW state were not used in the “between-session theta to alpha ratio” and the t-test. A Hanning window was applied for tapering. The frequency definition was set to 0.0–4.0 Hz for the delta wave, 4.0–8.0 Hz for the theta wave, 8.0–13.0 Hz for alpha wave, 13.0–30.0 Hz for beta wave, and over 30.0 Hz for the gamma wave. FFT was used to calculate the power of each EEG channel for all participants, and then the average value of each channel was calculated. Next, the theta to alpha ratio was calculated and compared for each channel. 

### 2.7. Statistics 

First, the power of theta and alpha was calculated for each participant. An EEG signal is not just a wave, but includes the power of the frequency of the wave. This can be found through fast Fourier Transform (FFT) [31]. FFT shows what power at which frequency and at what frequency the EEG has by time. FFT is a mathematical tool that describes the correlation between the time domain and the frequency domain of a wave [32]. Since the sample size was small, a normality test was performed with MATLAB (v9.5.0 R2018b, The MathWorks Inc.) to justify a statistical test for this value. Our statistical analysis protocol was as follows. Hypothesis: The theta/alpha index will be different between the Juingong meditation state and IMW state. The analysis model was based on a pair-wise t-test, two tail, and the alpha value was set to 0.05. We used the KS limiting form, KS Stephens modification, KS Marsaglia method, KS Lilliefors modification, Anderson–Darling test, Cramer–Von Mises test, Shapiro–Wilk test, Shapiro–Francia test, Jarque–Bera test, and the DAgostino and Pearson test to check whether the normality assumption is satisfied. The power of theta and alpha was calculated for the Juingong meditation and IMW state, respectively. This applied to all participants. The theta to alpha (T/A) ratio was calculated using the power values of theta and alpha obtained in this way. After this process, we obtained the T/A ratios for each of the Juingong meditation and IMW states, for all participants. The T/A ratio was calculated using the theta power value as the numerator and the alpha power value as the denominator. A paired t-test was performed on the obtained T/A ratios. The paired t-test (two-tailed) was performed under the assumption that the T/A ratio would differ between the Juingong meditation state and IMW state. We analyzed the correlation of the T/A ratio with the items ‘sugye’, ‘regul’, ‘angeo’, and ‘start’ by Pearson-r. The meaning of each item is as follows. ‘Sugye’ is similar to baptism. It is a ceremony in which the participant receives a Buddhist name (like a baptismal name). This item indicates the frequency of attending ‘sugye’ events. ‘Regul’ means the frequency of attending regular worship. Buddhists regularly have intensive training sessions. ‘Angeo’ indicates the frequency of participation in that period. ‘Start’ means the date when practitioners first came to the Hanmaum Seon Center.

## 3. Results

Since Juingong meditation focuses on the fundamental mind rather than just relaxation, it was predicted that the theta to alpha ratio would decrease because more alpha waves would be induced relative to the theta waves. In a previous study, these changes in the EEG were found in the occipital and temporal regions [20,23].

**Figure 3:**
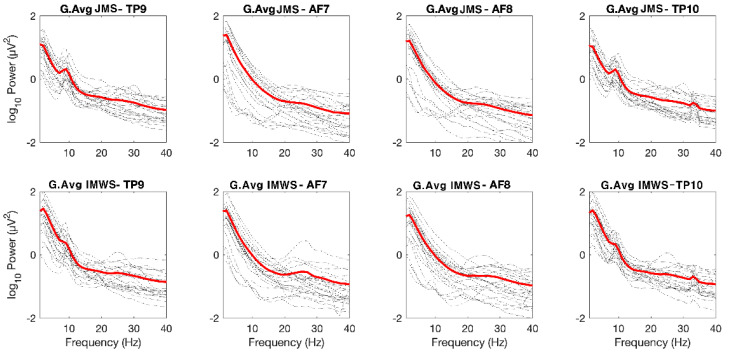
Frequency analysis grand average. G: grand, Avg: average. JMS: Juingong meditation state, IMWS: IMW state, Hz: Hertz TP: temporoparietal, AF: anterior frontal. In accordance with the international 10–20 system, the odd-numbered channel is placed on left side, the even-numbered channel is placed on the right.

**Figure 4:**
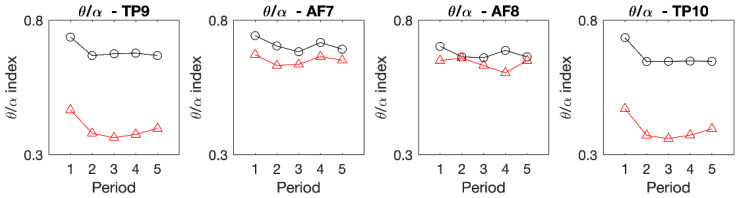
Within-session theta/alpha ratios by period. TP: Temporoparietal, AF: Anterior frontal, △: Juingong meditation, ○: IMW. The horizontal axis represents time, and the vertical axis represents the theta/alpha ratio. The total experimental time was 15 min, averaged for 3 min intervals, and displayed as a graph. The theta/alpha ratio is a value obtained by calculating the theta power and alpha power in each channel, and then using them as the numerator and denominator, respectively. In all areas, the theta/alpha ratio was lower in the Juingong meditation than in the IMW. The difference is particularly noticeable in the TP channels.

Figure 3 shows the results of performing FFT based on the original EEG data of each channel and converting this value to a logarithm for all participants. We obtained this data by first converting the EEG data obtained from the participants into FFT, and then converting it again using the log function. All four channels were divided into the Juingong meditation state and IMW state to analyze the EEG power by frequency. The black-dotted line is the individual EEG graph of each participant, and the red line is the average of the individual EEG power values of the participants (Figure 3). Figure 3 shows typical EEG characteristics for each channel. This graph pattern is similar to the normal EEG graph, in which the delta wave is the strongest, followed by the theta, alpha, beta, and gamma waves in order, which made it difficult to confirm any differences between the Juingong meditation state and IMW state. Therefore, the data measured for 15 min in the Juingong meditation state and IMW state were cut into 3-min intervals to obtain the theta to alpha ratio, and the average value was calculated (Figure 4). The Juingong meditation and IMW state were each considered as one session. Each participant took part in two sessions, one for Juingong meditation and one for the IMW state. One session represents EEG data measured for 15 min during the Juingong meditation and IMW state, respectively. We cut one session into 3 min intervals, and the EEG data measured for 3 min were expressed as one average. Five of these values were collected and are shown in Figure 4. One square box in Figure 4 represents one channel in which the EEG was measured for 15 min. Then, when the difference in each channel, which was not revealed in the grand average graph, was expressed by the theta to alpha ratio, it showed a marked difference. In Figure 4, red triangles represent the Juingong meditation state and black circles represent the IMW state. The horizontal axis represents time; the total EEG measurement time was cut into 3 min intervals and averaged. The vertical axis represents the T/A ratio. In all sections of all graphs, the Juingong meditation state showed a lower T/A ratio than the IMW state. In the AF channel, the difference in the T/A ratio was not clear, but in the TP channel, the difference was noticeable. 

**Figure 5:**
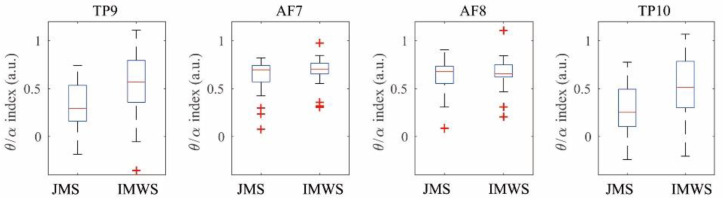
Between-session theta/alpha ratios paired t-test. JMS: Juingong meditation state, IMWS: IMW state, a.u.: arbitrary unit. The horizontal axis represents Juingong meditation state and IMW state. The vertical axis represents the theta/alpha ratio It can be seen that the theta/alpha ratio decreased in the JMS in the TP9 and TP10 channel. The theta/alpha ratio is a value obtained by calculating the theta power and alpha power in each channel, and then using them as the numerator and denominator, respectively. The theta/alpha ratio was also different in the AF channels, but the difference was smaller than that in the TP channels.

The difference in the TP channel was evident from periods 1 to 5. In this study, since the initial 5 min was taken as the time required to reach the concentration state, it was expected that the difference between the Juingong meditation state and IMW state would not be apparent at the beginning of the experiment. However, in the TP channel for the Juingong meditation state and IMW state, it was confirmed that the T/A ratio was constantly different from the beginning of the measurement to the end. The data for the first 5 min were discarded and the T/A ratio was calculated from the data for the second 10 min, as shown in Figure 5. The results of the paired t-test are shown in Table 1. As a result of the normality test for the T/A ratio data, the normality assumption was satisfied in the two TP channels (TP9 and TP10), but not in the two AF channels (AF7 and AF8). In TP9 and TP10, the average value of the T/A ratio of the Juingong meditation state was lower than that of the IMW state, and this difference was significant (*p* < 0.05). Finally, it was found that the average value of the T/A ratio of the Juingong meditation state was lower than that of the IMW state in the TP9 and TP10 channels, and this difference was significant (*p* < 0.05) (Figure 4, Table 1). Therefore, it was demonstrated that when Juingong meditation is practiced, the EEG pattern in the temporal lobe is different from the pattern during IMW.

**Figure 6:**
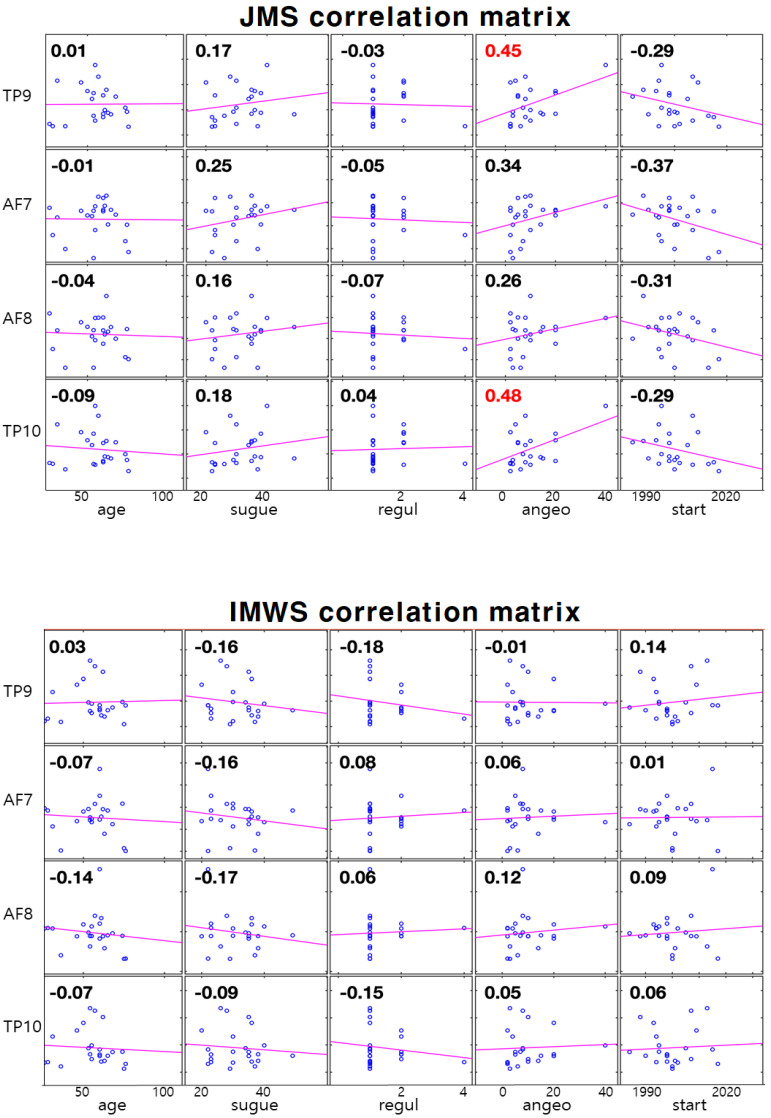
Correlation matrix between EEG channels (theta/alpha ratio of TP9, AF7, AF8, TP10) and questionnaire items. The top is JMS (Juingong meditation state), the bottom is IMW state correlation matrix. Sugye: frequency of attending ‘sugye’ events; regul: frequency of attending regular worship; angeo: frequency of participation of intensive training sessions; start: the date practitioners first came to the Hanmaum Seon Center. The purple line represents the trend line. The small blue circle in the box represents one practitioner. The number in each box represents the Pearson-r value.

As a result of analyzing the correlation between the T/A ratio and questionnaire items, most items were not significant. However, in the case of the Juingong meditation state, both TP9 and TP10 were found to be correlated with the ‘angeo’ category. However, this correlation did not appear in the IMW state. The correlation matrix for all items is shown in Figure 6 and the Pearson-r value indicating the correlation is shown in the Table 2.

## 4. Discussion

Since Juingong meditation is a form of meditation, we predicted that this would have similar characteristics to conventional meditation. Traditional meditation is mostly done in a sitting form. Juingong meditation does not have to be done sitting down. However, in order to obtain accurate experimental results, we asked participants to sit down during Juingong meditation. There are various types of meditation such as Vipassana, the Himalayan Yoga tradition, and the Isha Yoga tradition. When comparing the EEG data from these various meditation methods with each other, it is important to standardize the meditative form [6]. Therefore, we asked the participants to sit down and do the Juingong meditation. While there are various types of meditation, we expected that our results would be similar to those of the existing meditation EEG studies, just because the EEG was measured while sitting. The results of the experiment showed that the T/A ratio in the TP channels (TP9, TP10) was lower in the Juingong meditation state than in the IMW state. In the AF channels (AF7 and AF8), the Juingong meditation state showed lower average values than the IMW state, but the statistical tests were not significant, and the normality assumption was not satisfied. As a result of the review of previous studies, we found studies that did not use the data for the first few minutes of meditation for analysis because meditation is not performed immediately, only by maintaining a sitting position [6]. In regard to the participants’ opinions, according to the results of pre-interviews with Juingong meditation practitioners, many find it difficult to focus immediately at the beginning of meditation. Therefore, in this study, when comparing the t-test and between-session T/A ratio, the data for the first 5 min of the total EEG measurement time were discarded. The Juingong meditation and IMW are one session, respectively. Comparing between-session means was done by comparing the EEG data obtained from the Juingong meditation and IMW session. Figure 5 shows how the EEG from the Juingong meditation and IMW state is different according to each channel. However, looking at the within-session T/A ratios by period (Figure 4), it can be seen that the Juingong meditation state had a theta to alpha ratio different from that of the IMW state from the beginning. Since Juingong meditation and IMW are each regarded as one session, it is also important to find out how the T/A ratio changes during one session. One session is a recording of a 15-min EEG, which represents the within-session T/A ratios by period. In Figure 4, the average values of the T/A ratios for 3 min were collected 5 times and this shows what changes occurred during 15 min. This was applied to each EEG channel, and in Figure 4, one box represents one channel. These results were more apparent in the temporal parietal lobe than in the anterior frontal lobe. Therefore, it is thought that the decrease in the T/A ratio in the TP channels means that Juingong meditation induces relatively more alpha power, and does not only mean a decrease in the theta power. This can be interpreted as the result of increasing alpha power without reducing theta power. An increase in theta power is a phenomenon that occurs in conventional meditation because theta power increases in a deep relaxation state [33]. Alpha waves appear in more concentrated conditions than theta waves [34]. Alpha power is a marker of internal attention and it increases in concentration meditation practice [35]. If the participants had practiced conventional meditation, the theta/alpha ratio would have increased because the theta power would have increased rather than the alpha power. This is because the T/A ratio increases in a relaxation state rather than a concentration state [17]. However, in Juingong meditation, the T/A ratio decreased as a result. This can be interpreted as a result of the increase in alpha power rather than theta power. As a result of analyzing the correlation between the T/A ratio and the questionnaire items, in the case of the Juingong meditation state, both TP9 and TP10 were found to be correlated with the ‘angeo’ item. This means that the more you participate in intensive training, the clearer the relationship with the T/A ratio becomes.

Theta waves appear in a deep relaxation state, so they appear in hypnosis and meditation [33]. Alpha waves appear in more concentrated conditions than theta waves, but appear more often when concentrating in a quiet state rather than when moving around [34]. The lower T/A ratio in the Juingong meditation state can be seen as a result of it inducing more concentration than relaxation. The T/A ratio is derived from alpha to theta neurofeedback training. The result of increasing theta power is a common result not only in the Alpha–Theta protocol but also in existing meditation-related EEG studies [24]. In previous studies, in regard to the Alpha–Theta protocol there have been few examples of the use of EEG analysis techniques on people who meditate, rather than patients, and the Alpha–Theta protocol and EEG studies related to meditation have mainly used relaxation therapy [33].

There have been many studies to determine the levels of theta power or the level of theta power compared to alpha power [36]. Therefore, the results of this study have shown that the theta to alpha ratio can be used not only in clinical cases, but also as a tool to analyze meditation. EEG is an indirect indicator of the state of the brain, so it is true that there are difficulties in using it in research, but it is useful as a tool to identify and track changes in neuropsychiatric disorders such as panic attacks, alcoholism, depression, stroke, and Parkinson’s disease [37]. The brain waves of neuropsychiatric patients are characterized by low specific frequency bands, severe left–right asymmetry of brain wave power, and unstable brain waves [37]. In the next study, by conducting additional research on the neurophysiological control mechanism and the degree of psychological relief of participants, the fact that there was a significant difference in the theta to alpha ratio of TP9 and TP10 in the Juingong meditation state may provide a basis for applying Juingong meditation to various neuropsychiatric patients.

We did not recruit participants on the basis of a specific period of expertise. Better research results would have been obtained if participants were recruited based on a defined period of expertise, but due to the study’s quasi-experimental nature, this was unavoidable, and there may be limitations in the interpretation of the results. In addition, various factors (gender, age, practicing level, physical condition, mental stress, etc.) that influence the EEG measurements in this study were not considered in this analysis, which may lead to limitations in interpreting the study results.

## 5. Conclusions

Juingong meditation (Gwan) has the advantage of being a simple and easy-to-use technique with no time and place restrictions. The effect of Juingong meditation on brainwave patterns has not been explored yet. When Juingong meditation is practiced, the theta/alpha ratio changes without delay, which means that the practical effect of Juingong meditation on brainwave patterns is immediately apparent. Additional contextual studies of how participants felt or other biophysiological responses could increase its potential use in clinical diagnosis and treatment.

## Figures and Tables

**Table 1:** Differences in theta/alpha ratio for the two states by channel.

Channel	States	M ± SD	Paired T	*p*
TP9	JMS	0.31 ± 0.28	−3.73	0.001
	IMWS	0.55 ± 0.37		
AF7	JMS	0.63 ± 0.20		0.166
	IMWS	0.68 ± 0.16		
AF8	JMS	0.60 ± 0.21		0.347
	IMWS	0.65 ± 0.20		
TP10	JMS	0.30 ± 0.27	−4.22	<0.000
	IMWS	0.54 ± 0.33		

Note: JMS: Juingong meditation state, IMWS: IMW state, TP; temporoparietal, AF; anterior frontal. Theta/alpha ratio values for each channel and each state are shown.

**Table 2:** Pearson-r values between EEG channels and questionnaire items.

JMS					
**Pearson-r**	**Age**	**Sugye**	**Regul**	**Angeo**	**Start**
TP9	0.01	0.17	−0.03	0.45	−0.29
AF7	−0.01	0.25	−0.05	0.34	−0.37
AF8	−0.04	0.16	−0.07	0.26	−0.31
TP10	−0.09	0.18	0.04	0.48	−0.29
**IMWS**					
**Pearson-r**	**Age**	**Sugye**	**Regul**	**Angeo**	**Start**
TP9	0.03	−0.16	−0.18	−0.01	0.14
AF7	−0.07	−0.16	0.08	0.06	0.01
AF8	−0.14	−0.17	0.06	0.12	0.09
TP10	−0.07	−0.09	−0.15	0.05	0.06

JMS: Juingong meditation state, IMWS: IMW state, TP; temporoparietal, AF; anterior frontal. Pearson-r values between EEG channels (theta/alpha ratio of TP9, AF7, AF8, TP10) and questionnaire items are shown. Sugye: frequency of attending ‘sugye’ events; regul: frequency of attending regular worship; angeo: frequency of participation of intensive training sessions; start: the date practitioners first came to the Hanmaum Seon Center.

## Data Availability

The data presented in this study are available on request from the corresponding author.
